# Machine learning‐derived identification of prognostic signature for improving prognosis and drug response in patients with ovarian cancer

**DOI:** 10.1111/jcmm.18021

**Published:** 2023-11-23

**Authors:** Qing Huan, Shuchao Cheng, Hui‐Fen Ma, Min Zhao, Yu Chen, Xiaolu Yuan

**Affiliations:** ^1^ Shandong Key Laboratory of Reproductive Medicine, Department of Obstetrics and Gynecology Shandong Provincial Hospital Affiliated to Shandong First Medical University Jinan Shandong China; ^2^ Bidding Management Office The Second Affiliated Hospital of Shandong University of Traditional Chinese Medicine Jinan Shandong China; ^3^ School of Medical Management Shandong First Medical University Jinan Shandong China; ^4^ Mianyang Central Hospital, School of Medicine University of Electronic Science and Technology of China Mianyang Sichuan China; ^5^ School of Science Wuhan University of Technology Wuhan Hubei China; ^6^ Department of Pathology Maoming People's Hospital Maoming Guangdong China

**Keywords:** immunotherapy, machine learning, ovarian cancer, tumour microenvironment

## Abstract

Clinical assessments relying on pathology classification demonstrate limited effectiveness in predicting clinical outcomes and providing optimal treatment for patients with ovarian cancer (OV). Consequently, there is an urgent requirement for an ideal biomarker to facilitate precision medicine. To address this issue, we selected 15 multicentre cohorts, comprising 12 OV cohorts and 3 immunotherapy cohorts. Initially, we identified a set of robust prognostic risk genes using data from the 12 OV cohorts. Subsequently, we employed a consensus cluster analysis to identify distinct clusters based on the expression profiles of the risk genes. Finally, a machine learning‐derived prognostic signature (MLDPS) was developed based on differentially expressed genes and univariate Cox regression genes between the clusters by using 10 machine‐learning algorithms (101 combinations). Patients with high MLDPS had unfavourable survival rates and have good prediction performance in all cohorts and in‐house cohorts. The MLDPS exhibited robust and dramatically superior capability than 21 published signatures. Of note, low MLDIS have a positive prognostic impact on patients treated with anti‐PD‐1 immunotherapy by driving changes in the level of infiltration of immune cells. Additionally, patients suffering from OV with low MLDIS were more sensitive to immunotherapy. Meanwhile, patients with low MLDIS might benefit from chemotherapy, and 19 compounds that may be potential agents for patients with low MLDIS were identified. MLDIS presents an appealing instrument for the identification of patients at high/low risk. This could enhance the precision treatment, ultimately guiding the clinical management of OV.

## 1 INTRODUCTION

Ovarian cancer (OV) is the leading cause of mortality among malignant tumours affecting females.^1^ The standard treatment for OV involves bilateral salpingo‐oophorectomy and hysterectomy, followed by post‐operative platinum‐based chemotherapy.^2^, ^3^ Immune checkpoint inhibitors (ICIs) have significantly contributed to the management of various cancer types across a broad spectrum of diseases and have become a therapeutic option with great therapeutic potential.^4^ ICI monotherapy has demonstrated clinical efficacy in various tumour types. However, ICIs have not been granted approval for the therapeutic management of OV, primarily due to the limited efficacy of immunotherapeutic agents in OV patients.^5^ A preliminary study conducted on a cohort of 20 individuals diagnosed with platinum‐refractory OV revealed that nivolumab treatment yielded an overall response rate of 15%.^6^ Similarly, the overall response rate was 21% in patients with OV who had received first‐line chemotherapy and were given avelumab, and only 9% in patients with advanced disease.^7^ The tumour immune microenvironment varies between patients and tumour types, and these differences in composition may suggest different obstacles to anti‐tumour immunity, which can impact how patients respond to specific immunotherapies.^8^ Therefore, it is crucial to examine the heterogeneity of the ovarian population to determine the prognosis of patients with OV and identify those who are most likely to benefit from immunotherapy.

In light of the accelerated advancements witnessed in the realm of bioinformatics, a myriad of gene signatures with predictive potential have emerged.^9^, ^10^, ^11^ However, the use of inappropriate molecular biomarkers can delay the optimal timing of treatment and impose substantial social and economic burdens. To address this issue, several multi‐gene signatures based on specific pathways have been developed (necroptosis. sig, ferroptosis. sig, m6A. sig).^12^, ^13^, ^14^, ^15^ Nevertheless, the application of multigene expression signatures in clinical practice is limited due to their underutilization, utilization of inappropriate machine‐learning methods and lack of rigorous validation through additional large cohorts. To overcome these challenges, effective strategies include the development of new markers with better predictive performance or the establishment of a comprehensive rubric consisting of multiple predictive markers.

For the purpose of filling these gaps, a machine learning‐derived prognostic signature (MLDPS) was constructed using a combination of 101 machine‐learning algorithms in 2626 OV patients from 12 multicentre cohorts. We further validated the clinical applicability value of our signature as well as its robust performance for predicting prognosis by comparing it with 21 published signatures. The established MLDPS can stratify patients with OV and predict the outcome of immunotherapy. We further uncovered the potential mechanisms that underscore the manifestations of MLDPS and discerned prospective medicinal compounds for individuals with OV. This work can facilitate the prediction and the selection of OV individual and personalized immunotherapeutic.

## 2 METHODS AND MATERIALS

### 2.1 Data acquisition

The GEO database was searched using specific keywords to retrieve the mRNA expression data of OV: ‘ovarian cancer’ and ‘OV’. To ensure the quality of the collected data, the data set must contain the patient's prognostic information and have a valid sample size of not less than 50 patients. After the preliminary evaluation, we chose and obtained 11 profiles that contain important prognostic details [GSE49997 (*N* = 194), GSE51088 (*N* = 151), GSE53963 (*N* = 174), GSE63885 (*N* = 75), GSE73614 (*N* = 107), GSE9891 (*N* = 278), GSE13876 (*N* = 415), GSE140082 (*N* = 380), GSE17260 (*N* = 110), GSE26193 (*N* = 107) and GSE32062 (*N* = 260)].^16^, ^17^, ^18^, ^19^, ^20^, ^21^, ^22^, ^23^, ^24^, ^25^, ^26^ Transcriptome data and OV patient clinical information were also obtained from TCGA (*N* = 375) (Table 1).

**TABLE 1:** Summary of data sets used in the study.

Data sets	PMID	Platform	GPL	Region	Number of samples	Survival time (days)
*Ovarian cancer cohorts*						
GSE49997	22497737	ABI (V2)	GPL2986	Austria	194	30 ~ 1490
GSE51088	24368280	Agilent‐012097 (V2)	GPL7264	USA	151	30 ~ 6995
GSE53963	25269487	Agilent‐014850	GPL6480	USA	174	9 ~ 6048
GSE63885	27028324	HG‐U133_Plus_2	GPL570	Poland	75	103 ~ 4024
GSE73614	27016234	Agilent‐014850	GPL6480	USA	107	0 ~ 6000
GSE9891	18698038	HG‐U133_Plus_2	GPL570	Australia	278	0 ~ 6509
GSE13876	19192944	Operon human v3	GPL7759	Netherlands	415	30 ~ 7020
GSE140082	28159814	Illumina HumanHT‐12	GPL14951	Germany	380	1 ~ 1307
GSE17260	20300634	Agilent‐014850	GPL6480	Japan	110	30 ~ 2430
GSE26193	30244973	HG‐U133_Plus_2	GPL570	France	107	3 ~ 7280
GSE32062	22241791	Agilent‐014850	GPL6480	Japan	260	30 ~ 3840
TCGA‐OV	–	Illumina RNAseq	–	–	375	8 ~ 5481
*Immunotherapy cohorts*						
IMvigor210	29443960	Illumina RNAseq	–	USA	348	72 ~ 8934
Van Allen	26359337	–	–	Germany	42	34 ~ 1632
Nathanson	27956380	–	–	USA	64	73 ~ 2884

### 2.2 Data preprocessing

Subsequently, the gene expression data obtained by screening were cleaned, and the data of multiple probes corresponding to the same gene were averaged and combined. Using the R package ‘affy’, a robust multi‐array average algorithm was used for quantile normalization, background correction, and log_2_ transformation of raw data from the GEO database. The transcripts per million were obtained by converting the raw count of RNA‐seq data. To remove batch effects and ensure normalization, we utilized the R package ‘sva’ to process the merged expression matrix.^27^

### 2.3 Identify hub genes

We used univariate Cox analysis to detect prognosis genes. The common prognostic genes in the 12 data sets are known as stable OV prognostic genes. To explore differences in expression levels of genes between normal and tumour patients, this study used ‘limma’ packages to perform RNAs differential expression analysis based on data sets TCGA‐OV and GTEx.^28^ Statistically significant differentially expressed genes were determined based on RNAs having an adjusted *p*‐value < 0.05 and |log2FC| ≥ 1 among them.

### 2.4 Consensus clustering

Using K‐means algorithms, consensus clustering was applied to identify distinct patterns based on the risk genes‐related expression profile data. This process was implemented by the R package ‘Consensus Cluster’.^29^ To ensure that these categories were stable, 1000 iterations were conducted. Afterwards, the synthesis of the consensus score matrix and the application of the CDF curve were utilized to determine the most suitable quantity of clusters.^30^

### 2.5 Immune infiltration estimate

Using the R package ‘GSVA’, ssGSEA was conducted to quantify the relative infiltration abundance of immune cell populations and immune functions in the meta cohort.^31^ Seven other algorithms were applied to examine the stability and authenticity of the results of ssGSEA: Cibersort‐ABS, MCP‐counter, quanTIseq, TIMER, xCell, TIP and TISIDB.^32^

### 2.6 Enrichment analysis

To identify the potential biological roles and pathways between two clusters, we applied GO categories and KEGG enrichment analyses via the R package ‘clusterProfiler’. GSEA was further conducted to identify the potential regulating mechanism of MLDPS.^33^ The genes were ranked by log_2_FC following differential analysis using the ‘limma’ package.

### 2.7 Machine learning‐based integrative approaches‐derived MLDPS

**FIGURE 1:**
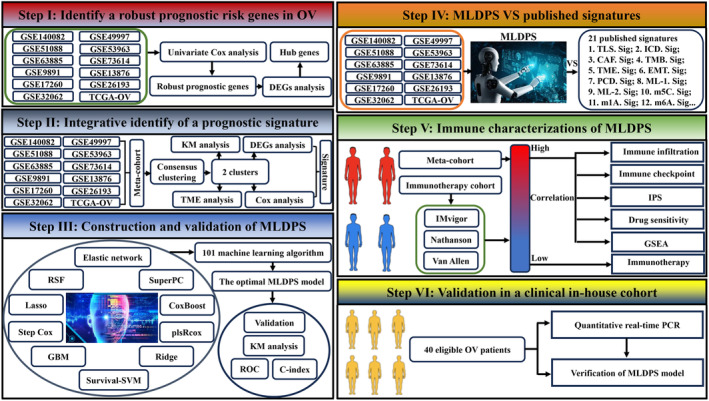
The workflow of this study.

The overall workflow of our study is presented in Figure 1. To develop an MLDPS that is highly accurate and stable, gene expression profiles across all data sets were converted into z‐scores to enhance comparability between different samples. Following is the procedure for generating signatures:

- DEGs analysis and univariate Cox regression analysis were applied to identify signatures between two clusters.
- Subsequently, the integration of 10 machine‐learning algorithms was conducted. Using 10‐fold cross‐validation approaches, the algorithms were randomly combined, and 101 combinational algorithms were generated.
- All these 101 combinations were conducted in the TCGA‐OV, GSE49997, GSE51088, GSE53963, GSE63885, GSE73614 (*N* = 107), GSE9891, GSE13876, GSE140082, GSE17260, GSE26193 and GSE32062 cohorts. The optimal signature was determined by calculating its average C‐index across all cohorts.

### 2.8 Retrieved published signatures for OV

A total of 21 published signatures from PubMed were included in the subsequent analysis (Table S1). Due to the insufficient data on miRNAs in our study participants, we did not gather miRNA signatures. Additionally, these signatures were constructed by an array of machine learning algorithms, for instance, stepwise Cox, LASSO, RSF, and GBM. Following this, we performed calculations of the C‐index to compare and contrast the MLDPS performance with that of other signatures.

### 2.9 Prediction of potential chemotherapeutic drugs

To assess dissimilarities in drug sensitivity between high and low MLDPS groups, we utilized RNA‐Seq data and drug sensitivity data from the GDSC database to generate drug sensitivity scores for each patient via the R package ‘pRRophetic’.^34^

### 2.10 qRT‐PCR

The 40 OV tissues were from the Department of Pathology of Maoming People's Hospital. This study was approved by the Ethics Review Board of the Maoming People's Hospital. All experiments complied with the relevant regulations, and all patients provided written informed consent. Total RNA was obtained from 40 OV tissues using Trizol reagent (catalogue number: R3901, Solarbio). According to the manufacturer's plan (SuperMix, catalogue number: R6734, Solarbio), reverse transcription into cDNA. Then, according to the manufacturer's protocol (SYBR GreenMaster Mix, catalogue number: R9303, Solarbio), and finally, further quantitative PCR was performed using the LightCycler96 real‐time fluorescence quantitative PCR instrument (catalogue number: 05815916001, Roche). The primer sequence is shown in Table S2.

### 2.11 Immunohistochemistry (IHC) staining

OV tissues were cut into 4‐mm serial sections. The tissue sections were heated at 60°C for l h, dewaxed with conventional xylene, dehydrated with gradient alcohol, and incubated at 37°C for 30 min in 3% H_2_O_2_ (Sigma). The sections were boiled with 0.01 M citrate buffer at 95°C for 20 min, cooled to room temperature and sealed with normal goat serum working solution for 10 min at 37°C. Next, the sections were probed with anti‐CD8 (1:200, CST), anti‐PD‐1 (1:200, CST) and PD‐L1 (1:200, CST) or equal doses of PBS as control at 4°C for 12 h and with biotin‐labelled goat anti‐rabbit secondary antibody (1:200, CST) for 10 min at ambient temperature. HRP‐labelled streptavidin working solution was supplemented dropwise for a 10‐min incubation at room temperature. The sections were developed with DAB at ambient temperature in the dark for 8 min, counter‐stained with haematoxylin (H8070, Solarbio Life Sciences Co., Ltd., Beijing, China), dehydrated, cleared, sealed and viewed under light microscopy.

### 2.12 Statistical analysis

This study is based on R (4.2.2) software for statistical analysis. Wilcoxon test was used for comparison of two groups, and Kruskal‐Wallis's test was used for comparison of multiple groups. In univariate and multivariate Cox regression analyses of genes, HR > 1 represents a risk factor for prognosis and HR < 1 represents a protective factor for prognosis. The statistical significance is defined as *p*‐value < 0.05.

## 3 RESULTS

### 3.1 Identify robust prognostic risk genes in OV

**FIGURE 2:**
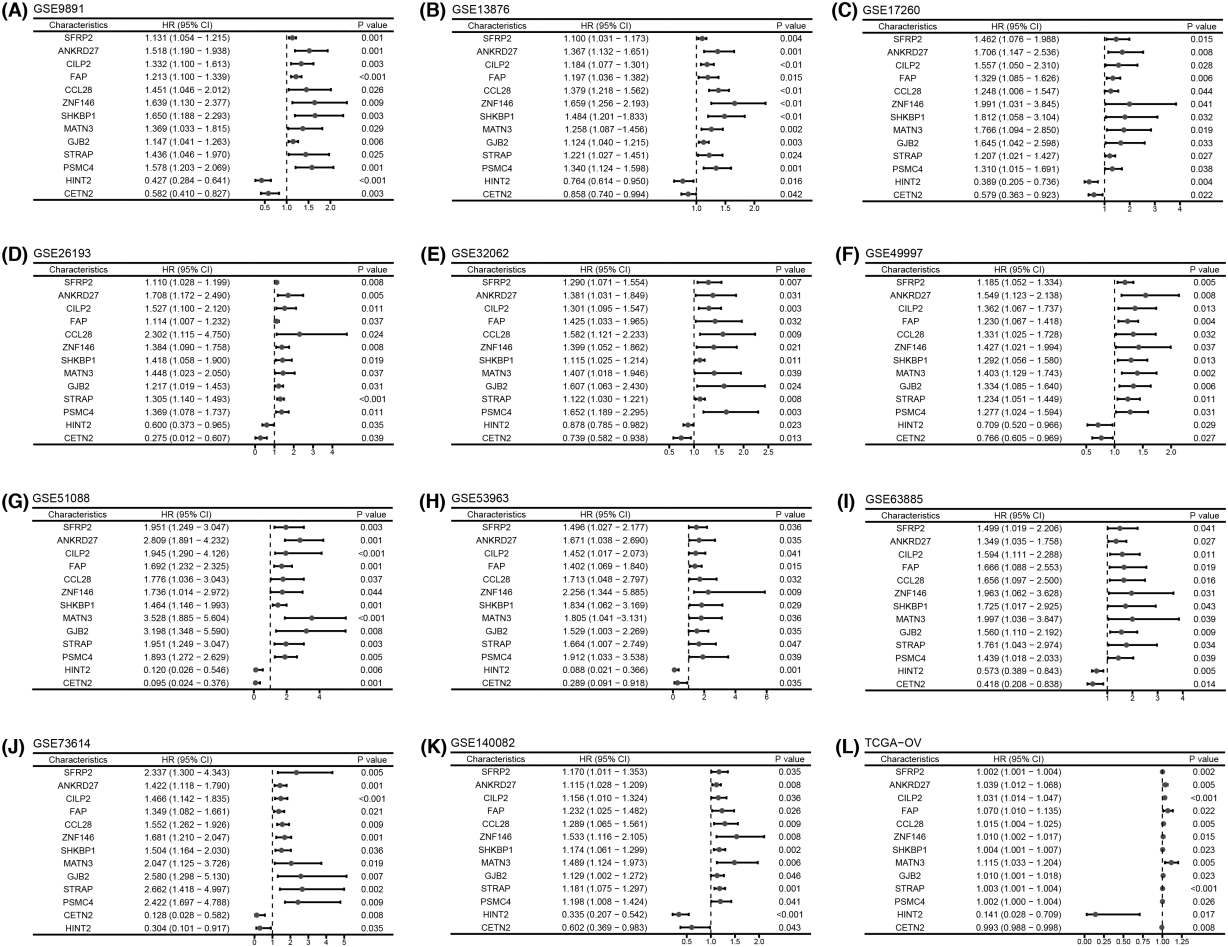
Identification of robust prognostic risk genes in ovarian cancer using multiple data sets. (A) GSE9891, (B) GSE13876, (C) GSE17260, (D) GSE26193, (E) GSE32062, (F) GSE49997, (G) GSE51088, (H) GSE53963, (I) GSE63885, (J) GSE73614, (K) GSE140082 and (L) TCGA‐OV.

Using univariate Cox regression analysis, 2463 prognostic genes were identified in GSE73614, 2043 prognostic genes were identified in GSE26193, 2121 prognostic genes were identified in GSE53963, 4136 prognostic genes were identified in GSE13876, 2372 prognostic genes were identified in GSE140082, 1859 prognostic genes were identified in GSE32062, 932 prognostic genes were identified in GSE17260, 2237 prognostic genes were identified in GSE51088, 1726 prognostic genes were identified in GSE63885, 2856 prognostic genes were identified in GSE9891, 1049 prognostic genes were identified in GSE49997 and 3933 prognostic genes were identified in TCGA‐OV (Table S3). After merging prognostic genes in 12 data sets, 13 robust OV prognostic genes were identified (Figure 2A–L). The findings also indicated that there was a remarkable elevation of 13 genes in OV tissues compared to the normal tissues obtained from GTEx (Figure S1). However, higher expression of two genes (CETN2, HNT2) had a better prognosis. Synthesizing the above information, 11 genes were identified as robust prognostic risk genes in OV.

### 3.2 Development and validation of consensus clusters based on 11 genes in a meta‐cohort

**FIGURE 3:**
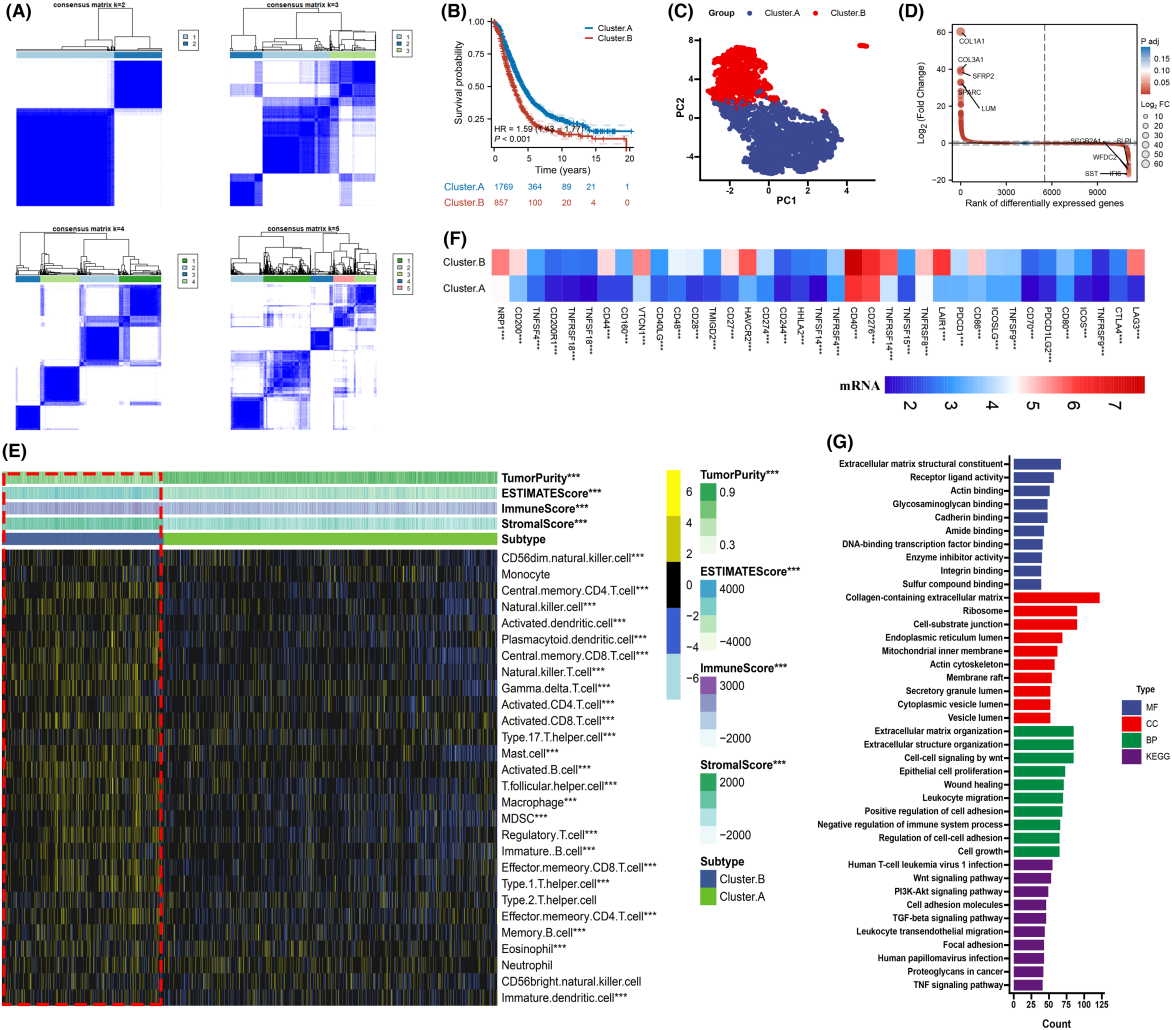
Development of consensus clusters based on 11 genes in a meta‐cohort. (A) The consensus score matrix of all samples when *k* = 2. (B) K‐M analysis of OS difference between the two clusters. (C) PCA analysis of difference between the two clusters. (D) DEGs between the two clusters. (E) The immune landscape between the two clusters. (F) The immune checkpoint expression between the two clusters. (G) Functional enrichment analysis of signature between the two clusters (*p < 0.05, **p < 0.01, ***p < 0.001).

First, we merge GSE49997, GSE51088, GSE53963, GSE63885, GSE73614, GSE9891, GSE13876, GSE140082, GSE17260, GSE26193, GSE32062 and TCGA‐OV into a meta‐cohort. Next, the initial division of all OV samples was performed, sorting them into *k* clusters using 11 genes within a meta‐cohort. The results of cluster analysis showed that *k* = 2 was the best cluster (Figure 3A). Kaplan–Meier analysis showed that cluster. A had better survival outcomes than cluster.B, and was validated in 12 cohorts (Figures 3B and S2A–L). Furthermore, a significant difference between the two clusters was observed (Figure 3C), and 1028 DEGs were identified between the two clusters (Figure 3D, Table S4). Next, the immune cell infiltration was lower in the cluster. A group, but the tumour purity was higher in cluster.A (Figures 3E and S3). Furthermore, according to the results, it was found that the cluster.B group exhibited notably higher expression levels of immune checkpoint compared to the cluster.A group (Figure 3F). To explore the potential biological change between distinct clusters, we explored prognostic genes, and 506 prognostic genes were identified (Table S5). Based on the GO and KEGG enrichment analysis results, it was evident that these specific genes demonstrated a remarkable enrichment towards the immune system, such as wound healing, leukocyte migration and TNF signalling pathway (Figure 3G). Based on the aforementioned findings, it is evident that the genes associated with clusters in the module exhibit significant enrichment in immune‐related pathways, suggesting that they play a key role in the progression of OV.

### 3.3 Integrative construction of MLDPS

**FIGURE 4:**
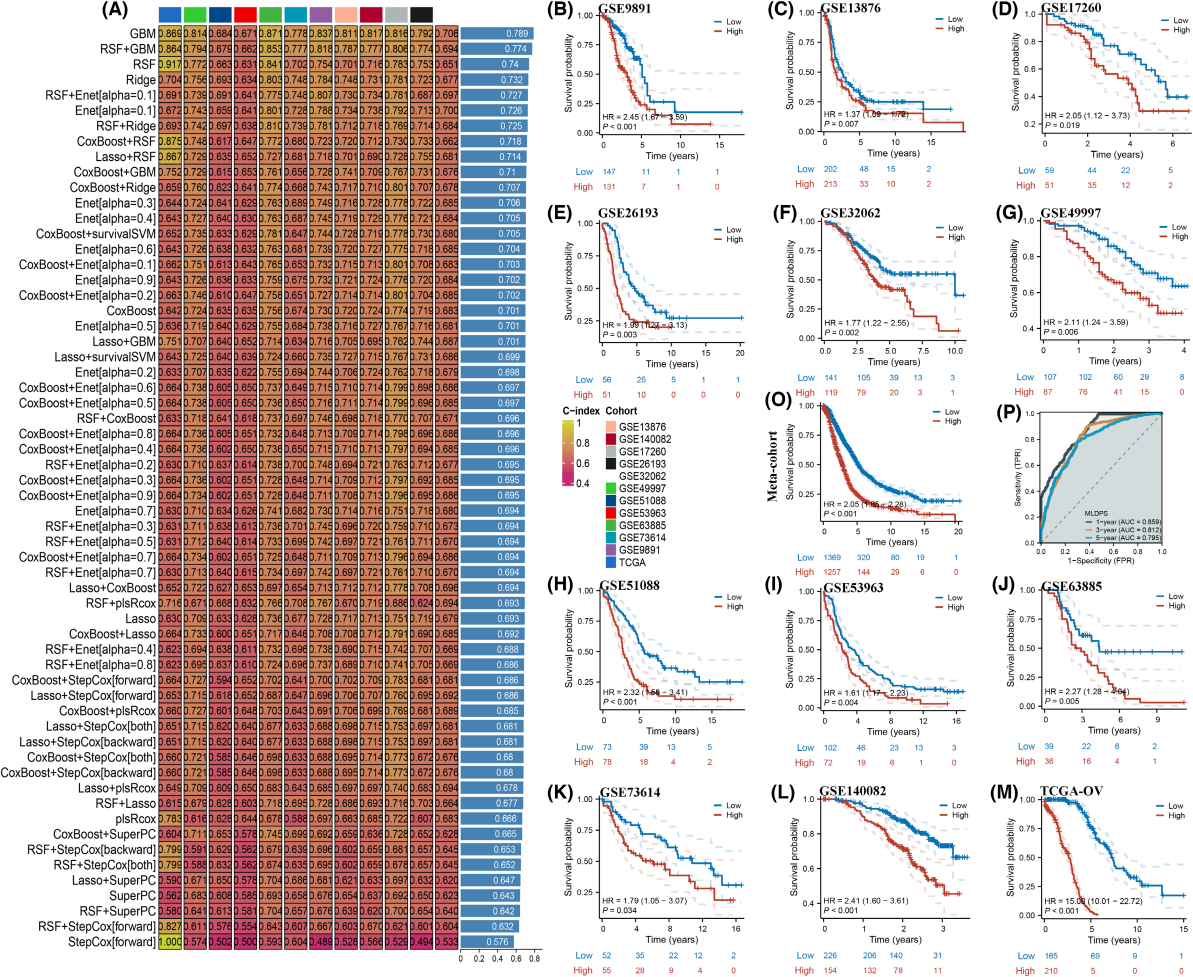
An MLDPS was developed and validated using multiple machine‐learning algorithms. (A) A total of 101 combinations of machine‐learning algorithms for the MLDPS via a 10‐fold cross‐validation framework. The C‐index of each model was calculated across 12 data sets. (B–O) Kaplan–Meier survival analysis of OS between the high and low MLDPS groups in GSE9891 (B), GSE13876 (C), GSE17260 (D), GSE26193 (E), GSE32062 (F), GSE49997 (G), GSE51088 (H), GSE53963 (I), GSE63885 (J), GSE73614 (K), GSE140082 (L), TCGA‐OV (M), meta (O). (P) Time‐dependent ROC curves of 1‐year, 3‐year and 5‐year OS in the meta‐cohort.

Next, an integrative approach based on machine learning was used to develop an MLDPS based on 506 prognosis‐related genes. As outlined in the methodology section, a fascinating observation revealed that the prime model happened to be GBM, establishing the peak average C‐index (0.789) across all validation data sets. This amalgamated model showcased unparalleled performance (Figure 4A). Next, using K‐M survival analysis, it was found that patients belonging to the high MLDPS category exhibited noticeably decreased overall survival (OS) compared to those in the low MLDPS group within the training cohort (TCGA‐OV, Figure 4M, *p* < 0.001) and other validation cohorts, including GSE9891 (Figure 4B, *p* < 0.001), GSE13876 (Figure 4C, *p* = 0.007), GSE17260 (Figure 4D, *p* = 0.019), GSE26193 (Figure 4E, *p* = 0.003), GSE32062 (Figure 4F, *p* = 0.002), GSE49997 (Figure 4G, *p* = 0.006), GSE51088 (Figure 4H, *p* < 0.001), GSE53963 (Figure 4I, *p* = 0.004), GSE63885 (Figure 4J, *p* = 0.005), GSE73614 (Figure 4K, *p* = 0.034) and GSE140082 (Figure 4L, *p* < 0.001). Furthermore, the meta‐cohort also showed the same pattern (Figure 4O, *p* < 0.001), and we executed an analysis of the ROC curve to assess the effectiveness of the MLDPS in the meta‐cohort. AUC achieved values of 0.859, 0.812 and 0.795 at the intervals of 1, 3 and 5 years, correspondingly (Figures 4P and S4A–L). As a result of the above findings, the MLDPS demonstrated superior stability and the capacity to extrapolate across multiple independent cohorts.

### 3.4 Comparisons between MLDPS and 21 published signatures in OV

**FIGURE 5:**
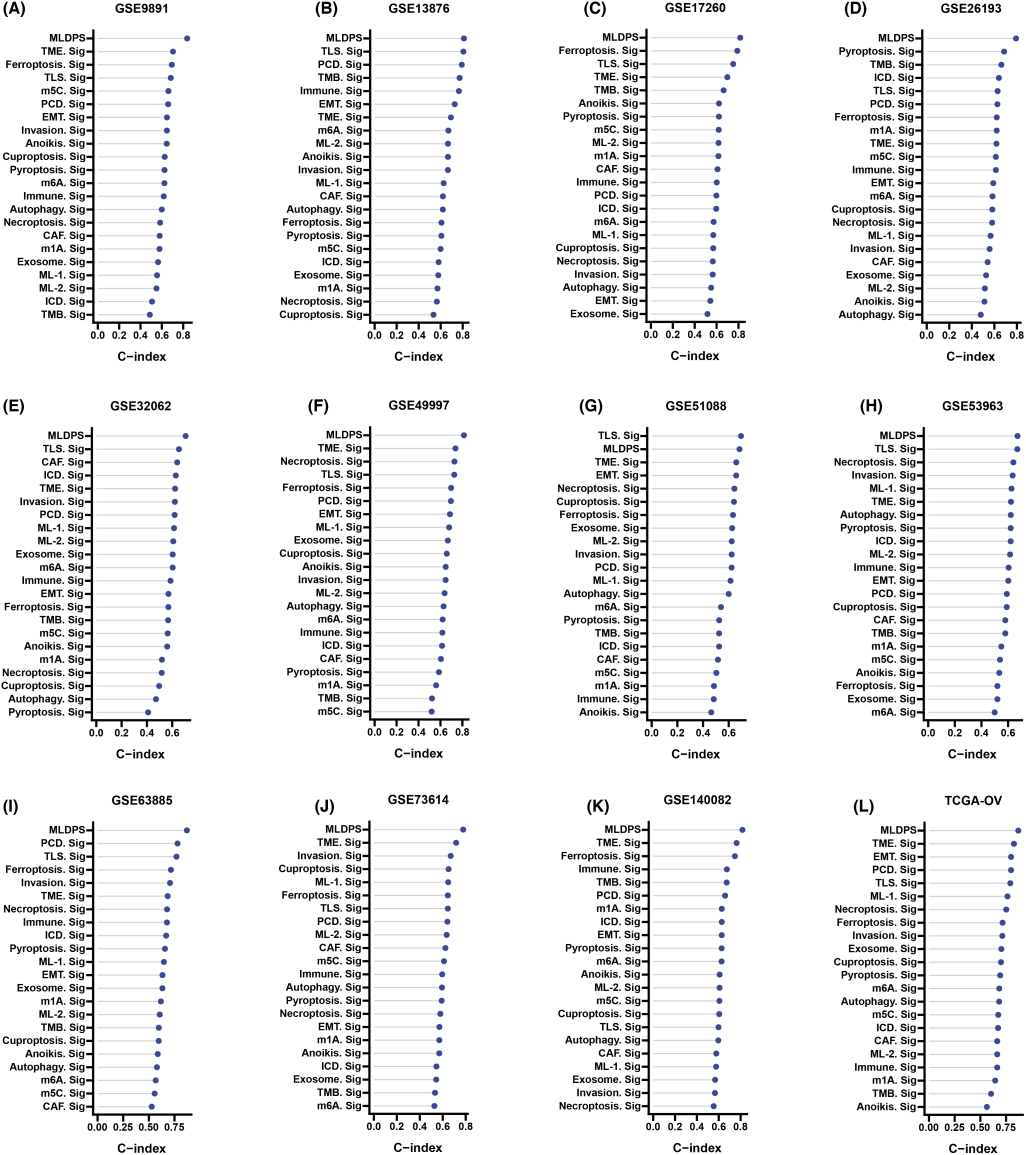
Comparison between the MLDPS signature and other OV‐related signatures. (A–L) The C‐index of the MLDPS signature and other 21 signatures developed in the GSE9891, GSE13876, GSE17260, GSE26193, GSE32062, GSE49997, GSE51088, GSE53963, GSE63885, GSE73614, GSE140082 and TCGA‐OV.

Machine‐learning algorithms have leveraged advances in next‐generation sequencing technologies and big data, enabling the development of prognostic and predictive gene expression signatures. Using the methods described above, we comprehensively retrieved published signatures to compare the performance of the MLDPS with other OV‐related signatures. Ultimately, 21 signatures were included in the subsequent comparisons (Table S1). These signatures were related to various biological processes, including necroptosis. sig, ferroptosis. sig, cuproptosis. sig, invasion. sig, pyroptosis. sig, TLS. sig, CAF. sig, m6A. sig, m5C. sig, TME. sig and other hotspots. The MLDPS obtained the highest C‐index (Figure 5A–L). In summary, the model prediction performance of the MLDPS far outperforms other signatures.

### 3.5 Enrichment analysis of MLDPS

**FIGURE 6:**
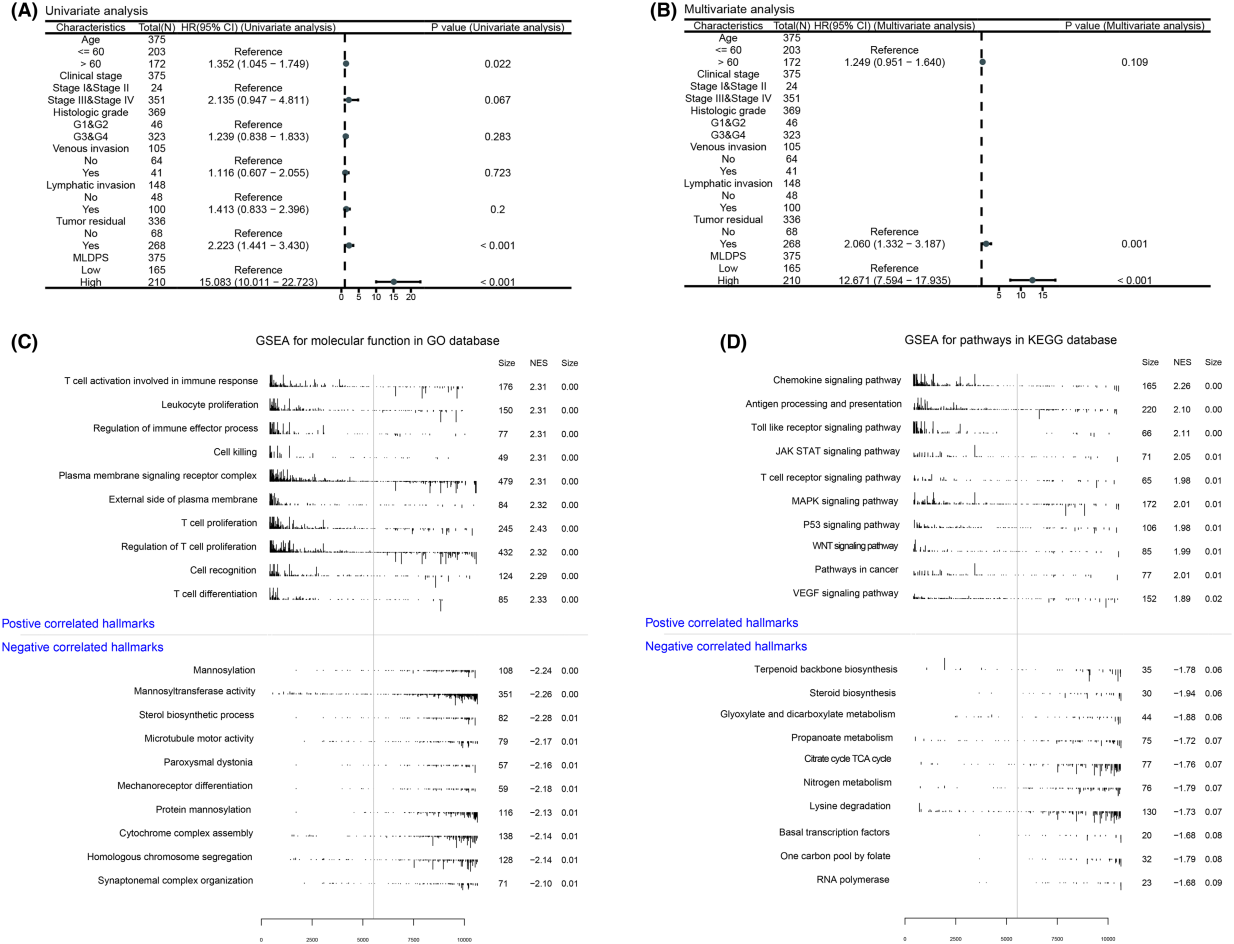
Pathway analyses of MLDPS in OV. (A–B) Results of the univariate (A) and multivariate (B) Cox regression analyses regarding MLDPS in the TCGA cohort. (C–D) Exploration of the potential pathways of MLDPS using GSEA GO (C) and GSEA KEGG analyses (D).

Over the past years, some clinical characteristics have exhibited a significant role in the assessment of prognosis risk and in the optimization of clinical decisions for patients with OV. To determine whether the MLDPS was a reliable prognostic indicator, we compared its performance to other clinical characteristics. As shown in Figure 6A,B, after adjusting with other clinical characteristics, including age, clinical stage, grade, venous invasion, lymphatic invasion and tumour residual, the prediction of OV patients' prognosis was effectively and consistently determined by the MLDPS.

Enrichment analysis was further applied to explore potential molecular and functional mechanisms for high and low MLDPS. As displayed in Figure 6C, high MLDPS were mostly enriched in T cell activation involved in immune response, leukocyte proliferation, T cell proliferation and T cell differentiation. KEGG analysis indicated high MLDPS were significantly enriched in T cell receptor signalling, JAK‐STAT signalling pathway, MAPK signalling pathway and VEGF signalling pathway (Figure 6D). The above results suggested the potential role of the MLDPS in OV tumorigenesis.

### 3.6 Immune landscape of MLDPS

**FIGURE 7:**
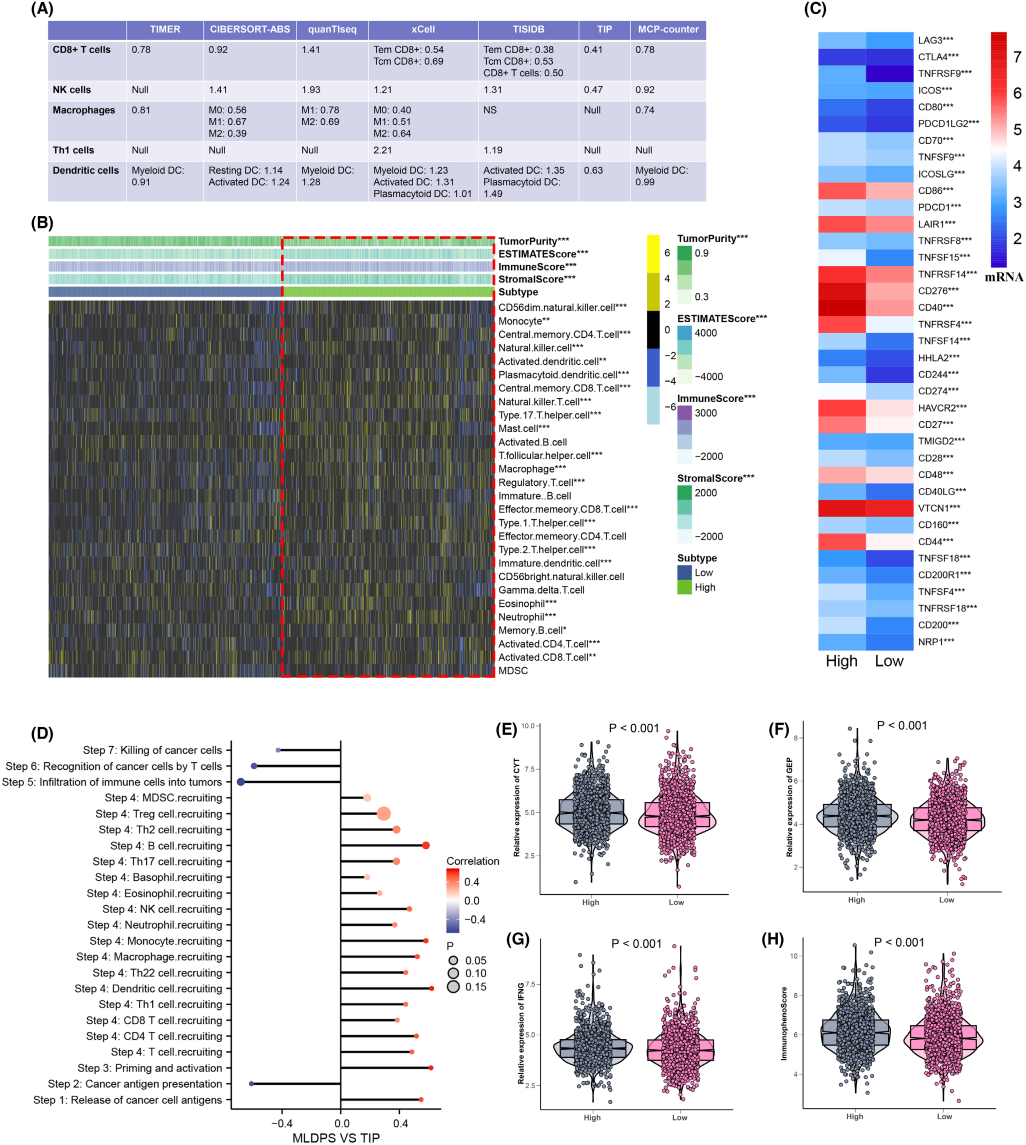
Immune landscape of MLDPS. (A) Correlations between MLDPS and the infiltration levels of five tumour‐associated immune cells. (B) The immune landscape between the high and low MLDPS. (C) The immune checkpoint expression between the high and low MLDPS. (D) Correlations between MLDPS and cancer immunity. (E‐H) Box plot displaying the CYT, GEP, IFN‐γ and IPS between high and low MLDPS groups (*p < 0.05; **p < 0.01; ***p < 0.001).

To explore the effect of MLDPS on levels of immune cell infiltration in the OV microenvironment, the correlation between MLDPS and immune cell infiltration was analysed by using seven independent algorithms. The results showed that MLDPS was positively correlated with the infiltration levels (Figure 7A). We also assessed the immune score and the stromal score in the high and low MLDPS groups and further added them together to obtain the estimated score using the ESTIMATE algorithm. In contrast to the low MLDPS group, the high MLDPS group demonstrated an elevated estimate score, with a substantial disparity observed between the two groups (Figures 7B and S5, *p* < 0.001). Furthermore, the results also showed that the expression level of immune checkpoint in the high MLDPS group was significantly higher than that in the low MLDPS group (Figure 7C). The results also suggest that the release of cancer antigen presentation, recognition of cancer cells by T cells, and killing of cancer cells were negative correlations with MLDPS. The findings suggest that while the low MLDPS group exhibited assistance in the initiation and processing stages of immune response, it still experienced a hindered effectiveness in generating antitumor immunity (Figure 7D). In order to promote the clinical availability of MLDPS, this study investigated the relationship between MLDPS and several immunotherapeutic predictors. Notably, the CYT, GEP, immunophenoScore and IFN‐γ levels were all significantly higher in the high MLDIS group (Figure 7E–H).

### 3.7 Immunotherapy response and drug response targeting MLDPS

**FIGURE 8:**
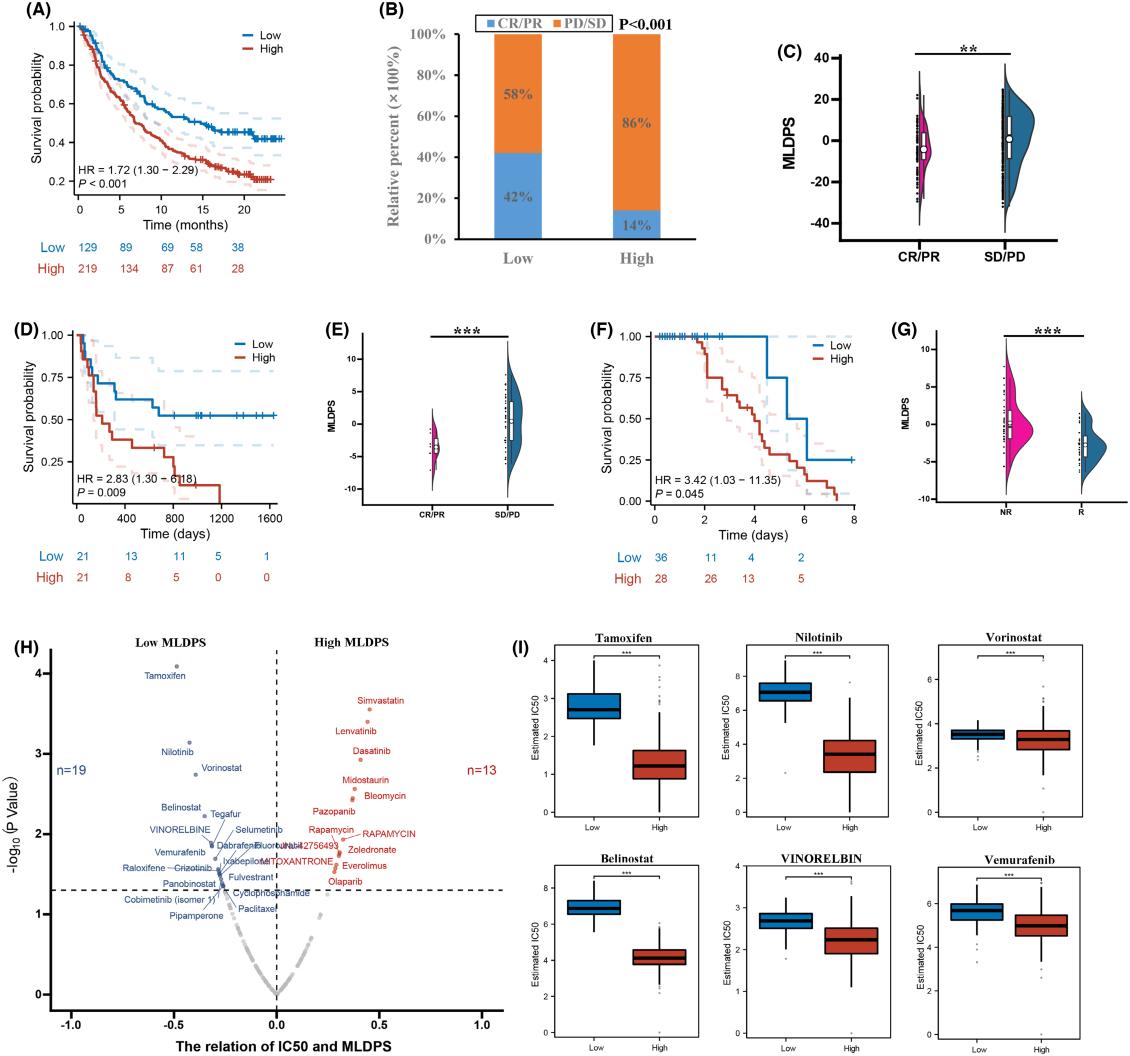
Predictive value of the MLDPS in immunotherapy response. (A) K‐M analysis of OS difference between the high and low MLDPS in the IMvigor data set. (B‐C) Box plot displaying the MLDIS in patients with different immunotherapy responses in the IMvigor data set. (D) K‐M analysis of OS difference between the high and low MLDPS in the Nathanson data set. (E) Box plot displaying the MLDIS in patients with different immunotherapy responses in the Nathanson data set. (F) K‐M analysis of OS difference between the high and low MLDPS in the Van Allen data set. (G) Box plot displaying the MLDIS in patients with different immunotherapy responses in the Van Allen data set. (H) The results of correlation analysis of derived compounds and MLDPS. (I) Distribution of the first six drugs in the high and low MLDPS (*p < 0.05, **p < 0.01, ***p < 0.001).

Based on the dissimilarities in immune traits between the two groups, the creation of the MLDPS is reliant on immune‐related patterns, we speculated that the sensitivity of OV patients with high/low MLDPS to immunotherapy will also be different. The results demonstrated that the lower MLDPS subgroup had longer survival time, and SD/PD group had a higher MLDPS than the CR/PR group in the IMvigor cohort (Figure 8A–C). Next, to further validate the robustness of MLDPS in immunotherapy in melanoma, the MLDIS model was constructed in two melanoma cohorts. According to the survival time analysis, it was determined that the subgroup with lower MLDPS exhibited a significantly extended lifespan in both the Van Allen and Nathanson cohorts. Moreover, the MLDPS exhibited a significant increase in the no‐response group compared to the response group in the Van Allen and Nathanson cohorts (Figure 8D–G). According to these results, immunotherapy was more likely to benefit the low MLDPS group. Next, we identified 19 GDSC‐derived drugs that were sensitive to patients in the low MLDPS group. The MLDPS group displayed a significant negative correlation with the estimated IC50 values of these agents, indicating higher values in this group (Figure 8H,I and S6).

### 3.8 Validation MLDPS in‐house cohort

**FIGURE 9:**
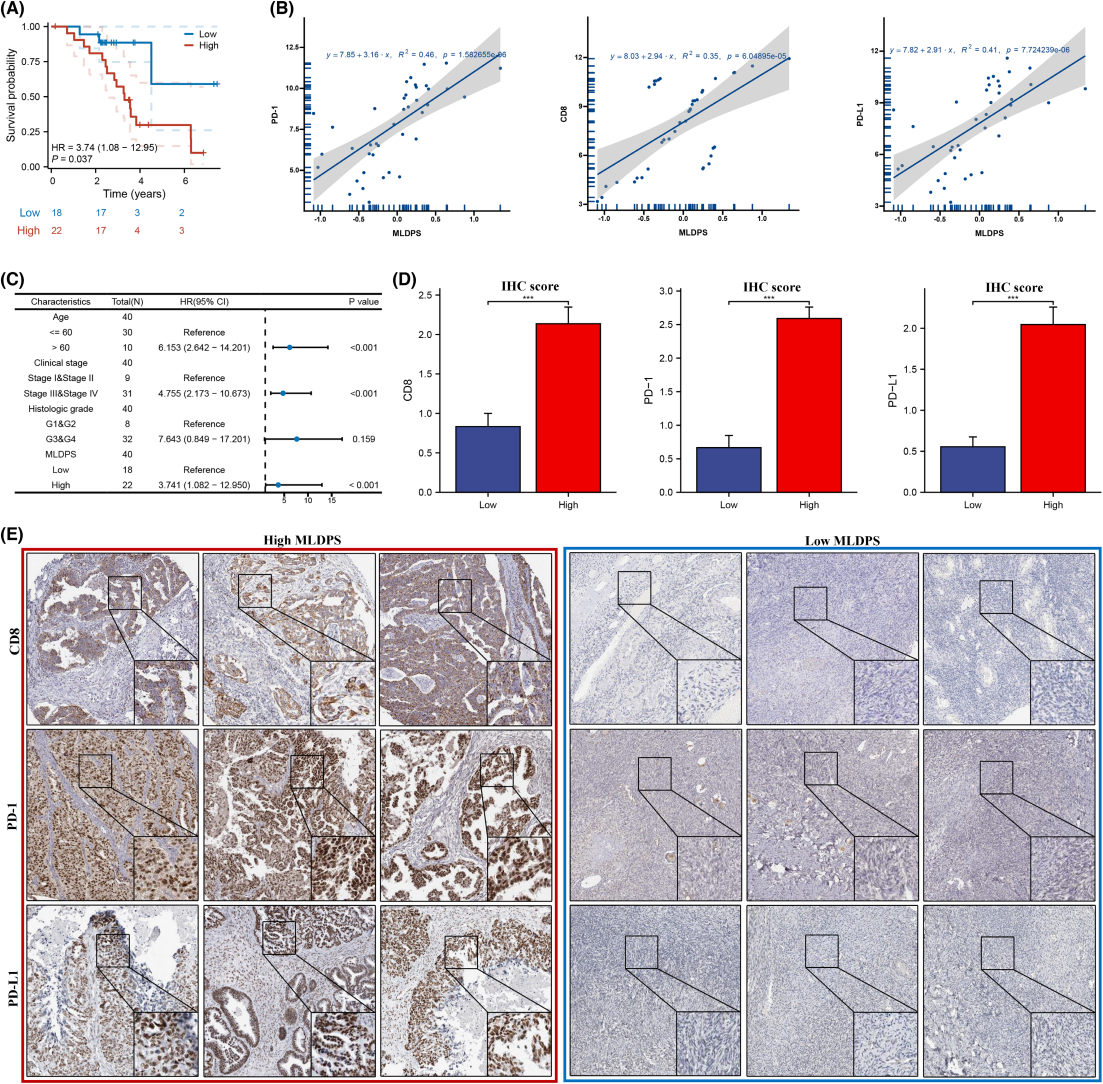
Validation in a clinical in‐house cohort. (A) K‐M analysis of OS difference between the high and low MLDPS. (B) Scatter plot displaying the correlation between the MLDIS and CD8, PD‐1 and PD‐L1. (C) Univariate Cox analysis of OS in the in‐house data set. (D) Box plot displaying the IHC score levels of CD8, PD‐1 and PD‐L1 based on IHC staining (E) Representative IHC staining images of CD8, PD‐1 and PD‐L1 in MLDIS groups (*p < 0.05, **p < 0.01, ***p < 0.001).

To further verify the performance of our MLDPS model in a clinically translatable tool, we next evaluated the expression of these RNAs in a clinical cohort of 40 OV patients by conducting qRT‐PCR assays. Consistently, according to the survival time analysis, it was determined that the subgroup with lower MLDPS exhibited a significantly extended lifespan in‐house cohort (Figure 9A,C). Subsequently, we inspected the correlation between the MLDPS and CD8, PD‐1 and PD‐L1 in the in‐house data set. The results demonstrated that MLDPS had a positive correlation with CD8, PD‐1 and PD‐L1 (Figure 9B). Moreover, CD8, PD‐1 and PD‐L1 were highly expressed in the high‐MLDIS group (Figure 9D,E).

## 4 DISCUSSION

OV, a highly malignant tumour of the genital tract, poses a significant medical challenge in terms of improving overall survival rates. While ICIs are not recommended as a standalone treatment for OV, there is growing interest in exploring the potential benefits of immunotherapy for OV patients. Recent research suggests that combining radiotherapy with immunotherapy can have a synergistic effect in controlling or eradicating cancer. Additionally, due to tumour heterogeneity and varying clinical outcomes among patients at the same stage, conventional clinical staging provides limited results for risk assessment and prognosis management.^35^ Recently, despite the widespread use of mutational biomarkers in clinical settings, their high cost, small‐scale and modest performance have hindered their better use in clinical settings.^36^ There is also the possibility of using liquid biopsy to determine prognosis and to guide treatment based on the circulating tumour DNA released by tumours, but further studies are needed to confirm this hypothesis.^37^ For clinical decisions to be effective, patients must be assessed individually due to the diversity of treatment options. Biological function signatures are derived from the expression data of multiple genes together using various machine‐learning algorithms and can be used for predicting OV prognosis. Nevertheless, deciding which algorithm to use and determining which algorithm is the best need deliberations. Individual preferences play a dominant role in influencing the choice of research included in the algorithm. This uncertainty leads to a lack of sufficient power for most established signatures in predicting patient prognosis and guiding clinical precision therapy. For the above reasons, establishing reliable prognostic biomarkers by using optimal integrative machine learning algorithms is required.

An innovative computational framework was used in this study in order to identify a robust and stable MLDIS. Firstly, we identified 11 robust prognostic risk genes in OV. The results of our analysis are in close agreement with the literature, which reports that 11 robust prognostic risk genes were expressed at different levels and have different biological functions in different tumours and the possibility of playing a specific role. For instance, chimaera of EpCAM aptamer and FAP siRNA obliterated intraperitoneal xenograft development of OV.^38^ TET1 potently inhibited canonical Wnt/β‐catenin signalling by demethylating and upregulating two upstream antagonists of this pathway, SFRP2 and DKK1, which was associated with inhibition of EMT and cancer cell metastasis.^39^ Secondly, based on 11 genes, we identified two OV subtypes, which have different phenotypes, a higher immune cell with higher immune infiltration but poor prognosis, and lower immune infiltration but better prognosis. Then, we screened out genes that differed and prognostic genes between the two OV subtypes. Finally, based on the above genes, we build MLDPS by 10 machine‐learning algorithms. Through integrative procedures, a model for OV prognosis can be fitted with consensus performance utilizing multiple machine learning algorithms. Subsequently results from K‐M analysis, ROC curve, and a comparison of clinicopathological features all indicated that the MLDPS had tremendous potential for clinical application based on its consistent performance in the multi‐cohorts. Moreover, a total of 21 published signatures of a variety of functional gene combinations were also retrieved and compared. As assessed using C‐index, the MLDPS outperformed in the comparison of predictive superiority among the published signatures. As a result, we believe that the MLDPS may be useful in evaluating OV prognosis in clinical settings.

According to the abovementioned results of the study, the MLDPS demonstrated superior stability in stratifying patients with high/low risk. Consequently, rational clinical intervention should be considered for patients with high/low MLDPS scores. Next, multiple algorithms revealed that the high MLDPS group was characterized by higher levels of immune cell populations and functions, such as CD4+ T cells, immune checkpoint expression and worse survival, which was conformity with the ‘immunity tidal model theory’.^40^ It is commonly acknowledged that these heightened cells with effector capabilities cannot bolster the immune response against tumours, thereby leading to the exacerbation of immunotherapeutic repercussions for individuals afflicted with elevated MLDPS. Scheper's team showed that only 10% of OV tumour microenvironment CD8(+) T cells were able to recognize tumour cells and that tumour‐reactive TCRs were absent from half of the patient tumour samples.^41^ Depletion of CD8(+) T lymphocytes may partly explain this phenomenon.^42^ In patients with tumour status where tumour antigen exposure continues to the immune system, T lymphocytes gradually lose effector function and up‐regulate the expression of inhibitory receptors, including PD‐1. T lymphocytes are depleted and upregulation of PD‐1 expression can be reversed by PD‐1 antibody therapy.

Immunotherapy is a promising treatment, but the response rate of OV to immunotherapy is not ideal. Investigating novel approaches to enhance the immunotherapy response rate among individuals with OV may represent a potential strategy, this is also the objective and focus of this study. In line with our conclusions, the results of TIP further confirmed that immunotherapy was more likely to be effective in patients with high MLDPS. In summary, the findings of our study indicated that the MLDPS offers a useful reference for identifying immunotherapy‐sensitive OV patients. In order to promote the implementation of precision medicine, it is crucial for healthcare professionals to promptly identify individuals with specific treatment sensitivities, thus enabling tailored interventions to meet their unique requirements. Platinum‐based doublet chemotherapy plays a crucial role in the treatment of advanced tumours, but unfortunately, drug resistance significantly reduces the chemosensitivity of chemotherapy, making drug treatment in patients with OV difficult.^43^ Therefore, we utilized the GDSC database, and comprehensive algorithms to develop specific drugs for patients with low MLDPS. We found that 19 chemotherapeutics such as tamoxifen, nilotinib, vorinostat and belinostat were more suitable for low MLDPS patients. Those FDA‐approved drugs may be potential candidate agents for low MLDPS patients and are being examined in ongoing clinical trials.

While the MLDPS is a promising comprehensive biomarker, this study has some limitations. It is imperative to understand in depth the molecular mechanisms that could influence the prognosis and immunotherapy response of OV mediated by MLDPS‐related genes. It is necessary to conduct further validation experiments in vivo and in vitro to clarify the function of most MLDPS‐related genes in OV. Second, some clinical and molecular features were not available in public datasets, resulting in a lack of research in this area, and this obscured the potential association between the MLDPS and certain variables. Finally, the validation of immunotherapy response using the MLDPS requires immediate attention, emphasizing the urgent need for cohorts of OV patients in immunotherapy studies.

## 5 CONCLUSION

In conclusion, we conducted an MLDPS model by using 10 machine‐learning algorithms (101 combinations). In addition to the expression of immune checkpoint genes, immune cell infiltrations in high and low MLDPS groups were also explored. Meanwhile, the prediction and selection of individual and personalized immunotherapeutic can be facilitated by the MLDPS model.

## AUTHOR CONTRIBUTIONS

**Qing Huan:** Conceptualization (equal); data curation (equal); writing – review and editing (lead). **Shuchao Cheng:** Formal analysis (equal); software (equal). **Hui‐Fen Ma:** Formal analysis (equal); software (equal); validation (equal). **Min Zhao:** Software (equal); validation (equal). **Yu Chen:** Methodology (equal); validation (equal). **Xiaolu Yuan:** Conceptualization (equal); writing – review and editing (equal).

## FUNDING INFORMATION

This work has received no funding.

## CONFLICT OF INTEREST STATEMENT

The authors have no conflict of interest to declare.

## Supporting information


**Supplementary Figure 1.** Differentially expressed module genes between tumour and normal tissue.

**Supplementary Figure 2.** (A–L) K‐M analysis of OS difference between the two clusters in the GSE9891, GSE13876, GSE17260, GSE26193, GSE32062, GSE49997, GSE51088, GSE53963, GSE63885, GSE73614, GSE140082 and TCGA‐OV.

**Supplementary Figure 3.** The differences in the infiltration of the immune cell populations in the two clusters.

**Supplementary Figure 4.** (A–L) Time‐dependent ROC curves of 1‐year, 3‐year and 5‐year OS in the GSE9891, GSE13876, GSE17260, GSE26193, GSE32062, GSE49997, GSE51088, GSE53963, GSE63885, GSE73614, GSE140082 and TCGA‐OV.

**Supplementary Figure 5.** The differences in the infiltration of the immune cell populations in the high and low MLDPS.

**Supplementary Figure 6.** Distribution of the other 13 drugs in the high and low MLDPS.

**Supplementary Table 1.** Retrieved 21 published signatures for OV.

**Supplementary Table 2.** The primer sequence used in this study.

**Supplementary Table 3:** The prognostic genes of each data set used in this study.

**Supplementary Table 4:** The different expression genes between the two clusters.

**Supplementary Table 5:** The prognostic genes in the clusters.

## Data Availability

The data sets generated for this study can be found in the GEO database (GSE49997, GSE51088, GSE53963, GSE63885, GSE73614, GSE9891, GSE13876, GSE140082, GSE17260, GSE26193, GSE32062, Van Allen and Nathanson; https://www.ncbi.nlm.nih.gov/geo/) and UCSC Xena website (https://gdc.xenahubs.net).
