# Supplementary figures and images for: Machine learning‐derived identification of prognostic signature for improving prognosis and drug response in patients with ovarian cancer
Source: J Cell Mol Med. 2023 Nov 23;28(1):e18021. doi: 10.1111/jcmm.18021 (PMC10805490; doi:10.1111/jcmm.18021)

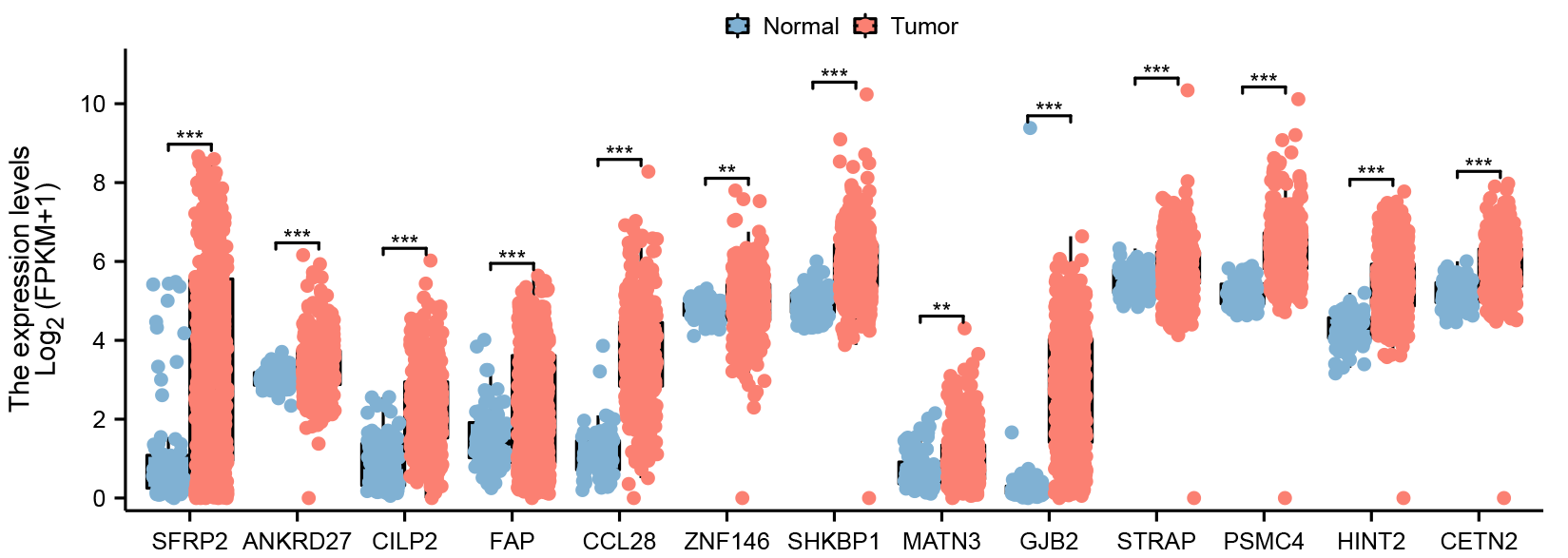

Supplement: Supplementary file 1 — Supplementary Figure 1. Differentially expressed module genes between tumour and normal tissue. [file JCMM-28-e18021-s006.tif]

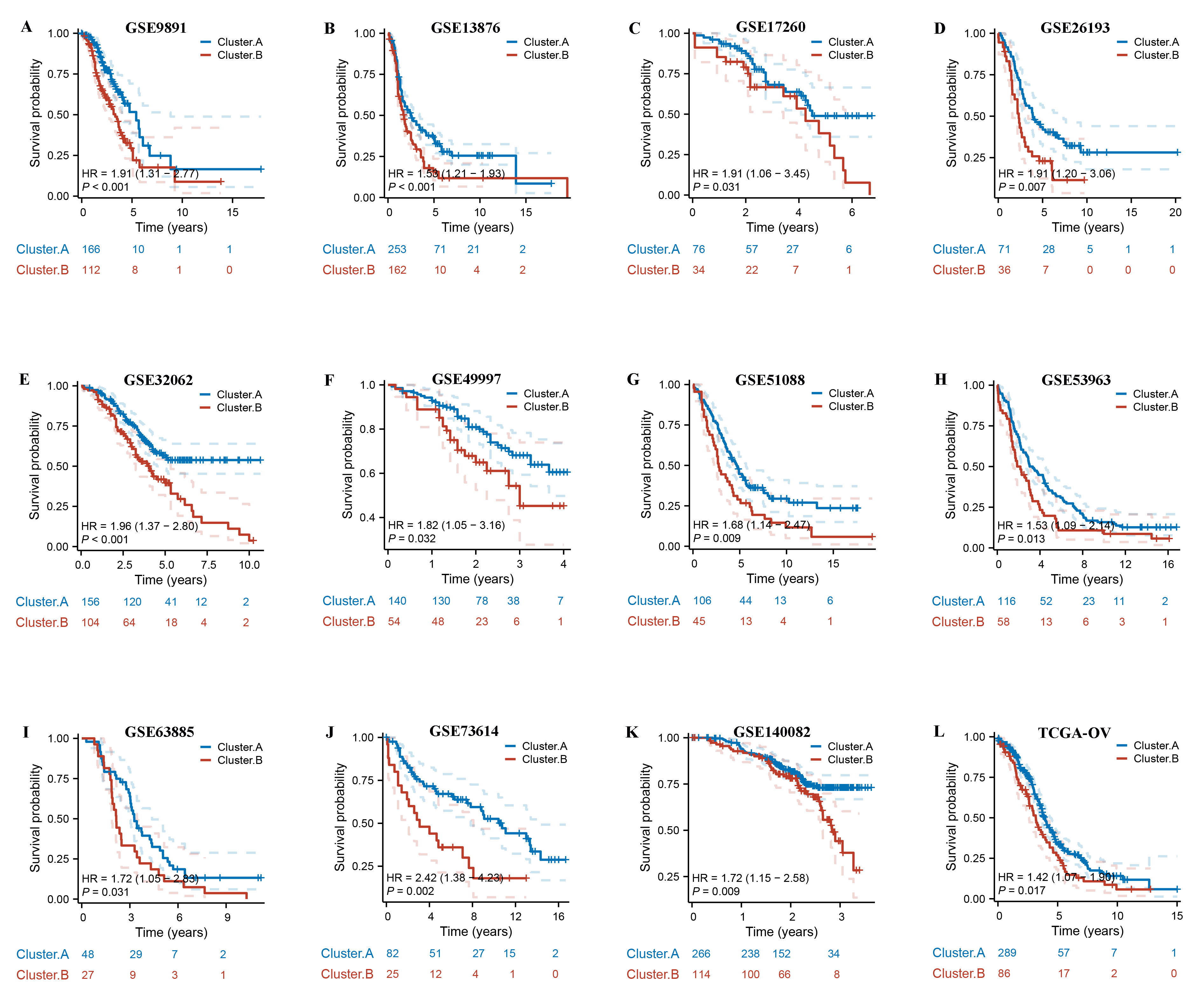

Supplement: Supplementary file 2 — Supplementary Figure 2. (A–L) K‐M analysis of OS difference between the two clusters in the GSE9891, GSE13876, GSE17260, GSE26193, GSE32062, GSE49997, GSE51088, GSE53963, GSE63885, GSE73614, GSE140082 and TCGA‐OV. [file JCMM-28-e18021-s007.tif]

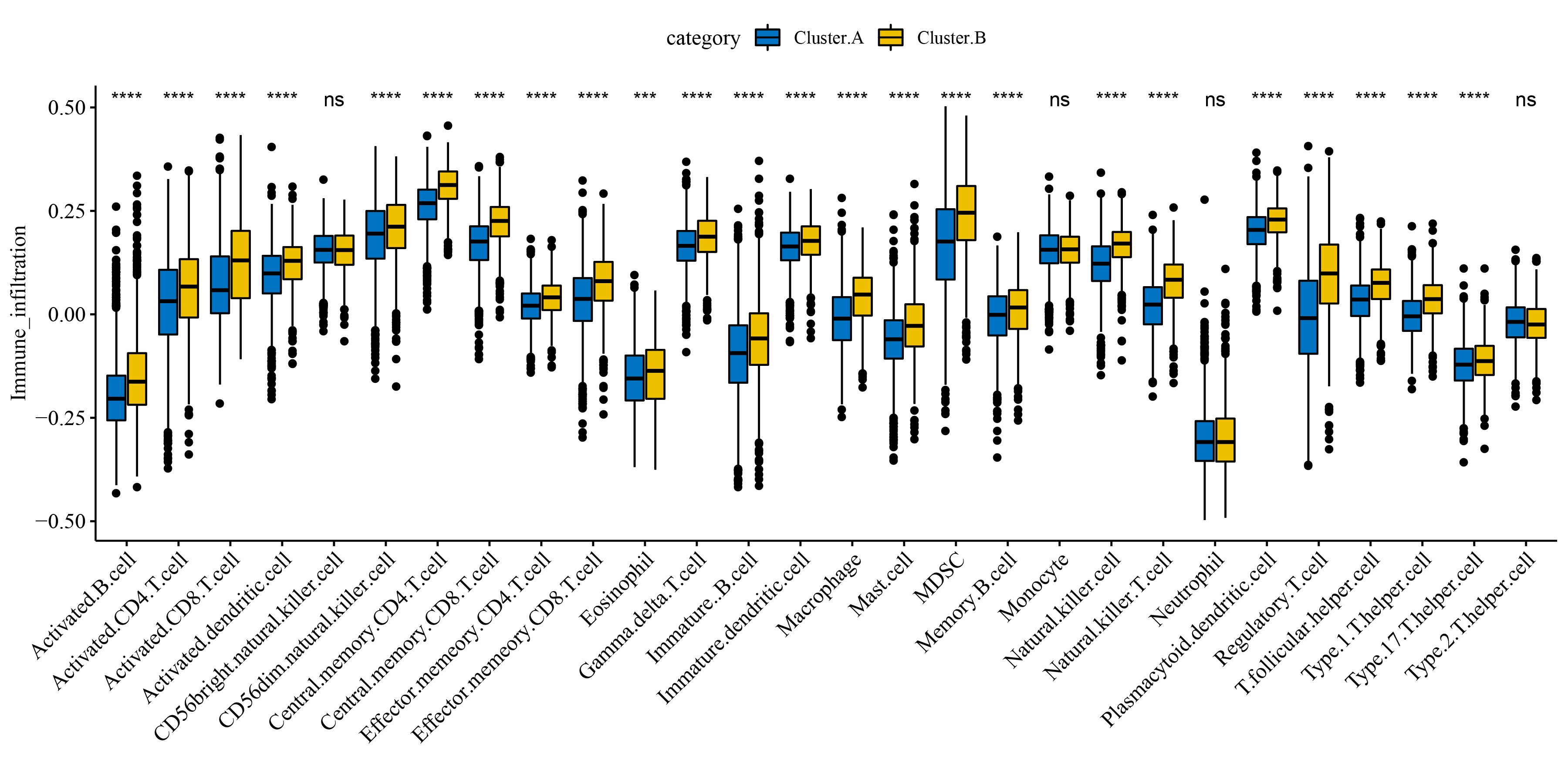

Supplement: Supplementary file 3 — Supplementary Figure 3. The differences in the infiltration of the immune cell populations in the two clusters. [file JCMM-28-e18021-s001.tif]

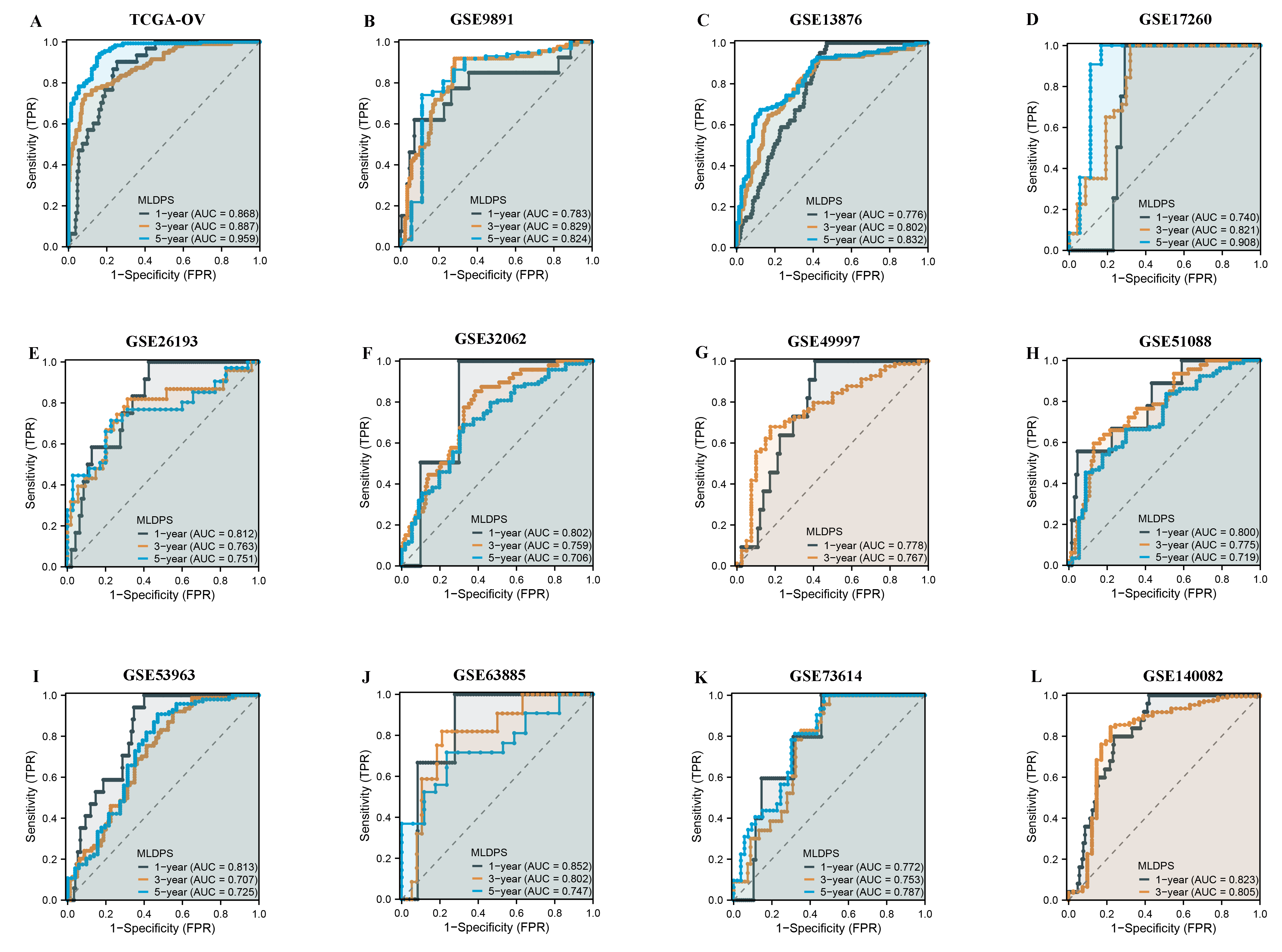

Supplement: Supplementary file 4 — Supplementary Figure 4. (A–L) Time‐dependent ROC curves of 1‐year, 3‐year and 5‐year OS in the GSE9891, GSE13876, GSE17260, GSE26193, GSE32062, GSE49997, GSE51088, GSE53963, GSE63885, GSE73614, GSE140082 and TCGA‐OV. [file JCMM-28-e18021-s004.tif]

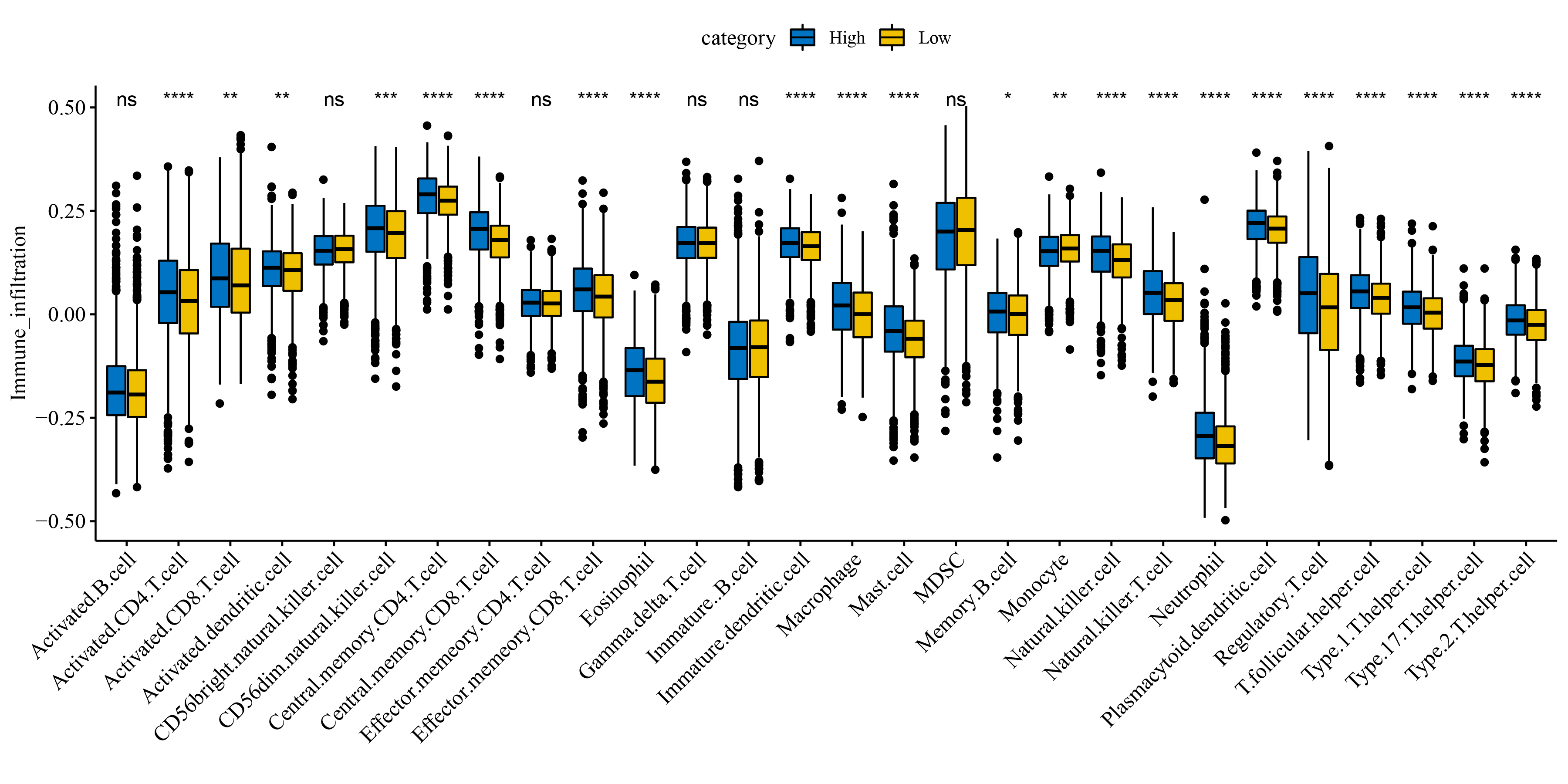

Supplement: Supplementary file 5 — Supplementary Figure 5. The differences in the infiltration of the immune cell populations in the high and low MLDPS. [file JCMM-28-e18021-s005.tif]

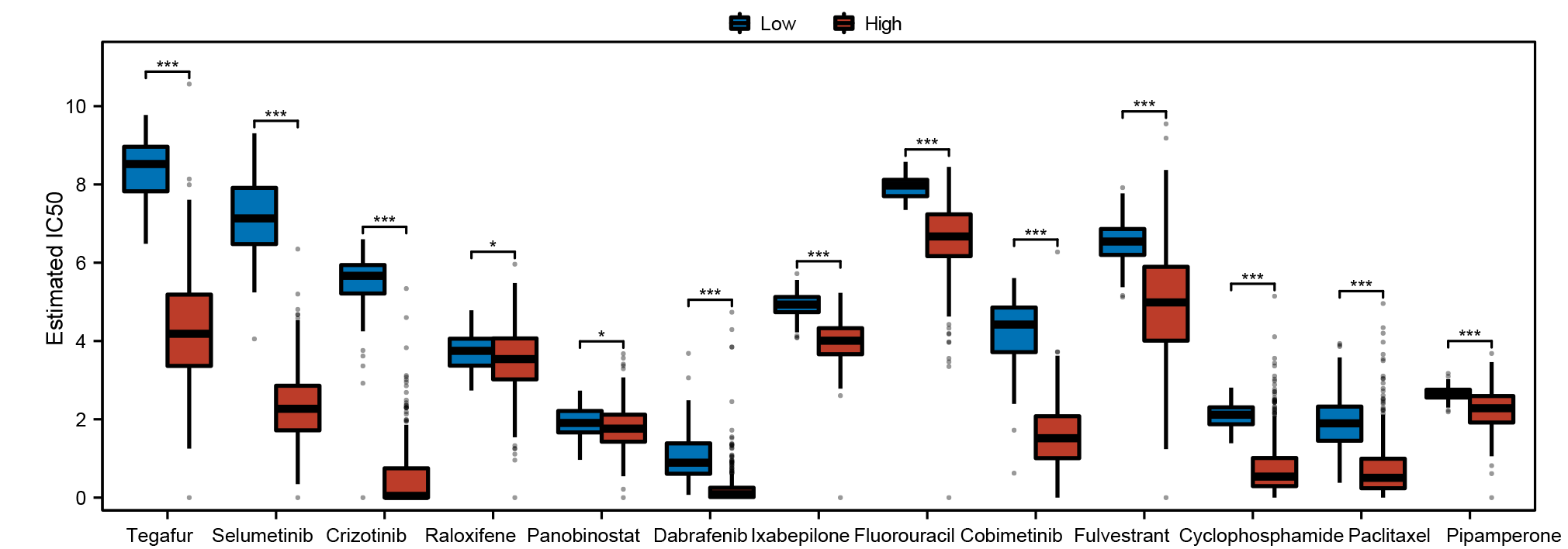

Supplement: Supplementary file 6 — Supplementary Figure 6. Distribution of the other 13 drugs in the high and low MLDPS. [file JCMM-28-e18021-s002.tif]
